# The Influence of Emulsifiers on the Physiochemical Behavior of Soy Wax/Rice Bran Oil-Based Oleogels and Their Application in Nutraceutical Delivery

**DOI:** 10.3390/gels9010047

**Published:** 2023-01-06

**Authors:** Somali Dhal, Abdullah Alhamidi, Saeed M. Al-Zahrani, Arfat Anis, Kunal Pal

**Affiliations:** 1 Department of Biotechnology and Medical Engineering, National Institute of Technology Rourkela, Rourkela 769008, India; 2 SABIC Polymer Research Center (SPRC), Department of Chemical Engineering, College of Engineering, King Saud University, Riyadh 11421, Saudi Arabia

**Keywords:** oleogel, wax crystals, soy wax, curcumin

## Abstract

This research evaluated the influence of stearic acid, sunflower lecithin, and sorbitan monooleate on soy wax (SYW)/rice bran oil (RBO)-based oleogels. The physiochemical behavior of oleogel samples was evaluated using colorimetry, microscopy, FTIR, mechanical, crystallization kinetics, X-ray diffraction, and a drug release investigation. The prepared oleogels were light yellow, and adding emulsifiers did not change their appearance. All oleogels showed an oil binding capacity of >98%, independent of emulsifier treatment. The surface topography revealed that emulsifiers smoothed the surface of the oleogels. Bright-field and polarized micrographs showed the presence of wax grains and needles. FTIR spectra indicated that oleogel samples had the same functional group diversity as the raw materials. The oleogel samples lacked a hydrogen-bonding peak. Hence, we postulated that non-covalent interactions were involved in the oleogel preparation. According to stress relaxation studies, the firmness and elastic component of oleogels were unaffected by emulsifiers. However, EML3 (oleogel containing sorbitan monooleate) showed lower relaxing characteristics than the others. EML3 exhibited the slowest crystallization profile. Due to its low d-spacing, EML3 was found to have densely packed crystal molecules and the largest crystallite size. The in vitro drug release studies showed that emulsifier-containing oleogels dramatically affected curcumin release. These results may help customize oleogels properties to adjust bioactive component release in the food and pharmaceutical industries.

## 1. Introduction

The word “nutraceutical,” coined by Stephen DeFelice in the year 1989, has been derived from the contraction of the words “nutrition” and “pharmaceutical.” An official definition of a nutraceutical is “a food (or portion of a food) that delivers medical or health advantages, including the prevention and/or treatment of illness” [1,2]. Such types of food products can range from vitamins (e.g., vitamin E, vitamin D, riboflavin, etc.), functional foods (e.g., bioactive peptides, prebiotics, probiotics, bioflavonoids), isolated nutrients (e.g., folic acid, lutein, etc.) [3], and genetically engineered foods (e.g., genetically engineered rice and genetically engineered banana). Many nutraceuticals have low chemical stability, low solubility, and low bioavailability. Therefore, researchers have formulated different delivery strategies to enhance the bioavailability and therapeutic efficacies of nutraceuticals. Various delivery systems, such as nanoparticles, microparticles, liposomes, hydrogels, oleogels, emulsions, phospholipid complexes, etc., have been studied and characterized for nutraceutical delivery [4]. However, limited studies are available on oleogel-based nutraceutical delivery systems among delivery vehicles.

Oleogels are semi-solid and non-crystalline systems of oil (e.g., canola oil, sesame oil, sunflower oil, hazelnut oil, soybean oil, olive oil, and rice bran oil). Herein, the oil forms the liquid phase. The oil phase is entrapped within the voids of the 3D networks formed via physical interactions (e.g., hydrogen bonding, van der Waals interaction, or π-stacking interaction) of the solid component called oleogelators [5,6,7]. The oleogels can significantly improve the stability of the bioactive molecules in the physiological environment by encapsulating the bioactive molecules in the 3D network. The oleogels help to isolate the bioactive molecules from the environment oxygen, thereby minimizing the oxidation of the bioactive molecules. The lipolysis of the encapsulated hydrophobic molecules is also markedly reduced. The reasons above can explain the improved stability of the bioactive molecules when incorporated within the oleogels. Further, the bioactive molecules can be released at a controlled rate, thus increasing their bioavailability [8]. Though many researchers have used oleogels as a carrier for vitamins, polyphenols, and volatile aromatic compounds, the research on the delivery of nutraceuticals using oleogels has not been widely explored. Recently, a few studies have demonstrated the efficacy of various oleogels for delivering lipid-soluble (or hydrophobic) nutraceuticals. For example, Calligaris et al. (2020) developed sunflower oil-based oleogels using different oleogelators for curcuminoid delivery. They found that the bioavailability of curcuminoid compounds might be modulated by the oleogelator’s ability to regulate lipolysis. Another group of researchers developed ethylcellulose/corn-oil-based oleogels by adding sorbitan monopalmitate as a surfactant to improve the solubility and stability of curcumin [9]. The addition of surfactant modified the interactions between the gelator and the oil phase by forming a compact gel system. Such a system slowed lipid digestion and minimized the formation of curcumin crystals during storage.

Emulsifiers (e.g., stearic acid, stearyl alcohol, SPAN60, TWEEN80) can alter the structural and mechanical properties of the oleogels. The properties of various wax-based oleogels have been enhanced using emulsifiers [10,11,12]. Depending on the length of the hydrocarbon chains and functional groups, the emulsifiers exhibit specific properties. The chemical nature (or polarity) of the head and tail groups of the emulsifiers can affect the sol-to-gel transition temperatures [10]. Hence, emulsifiers can alter the gel elasticity and viscosity [13]. They also have the potential to modify the crystalline structures by affecting their lipid polymorphism during gelation. Therefore, emulsifiers have also been called “crystal modifiers” by many authors. However, a comparative study on different emulsifiers is yet to be carried out to identify the effect of emulsifiers on the properties of oleogels.

In the current study, we have synthesized soy wax (SYW) and rice bran oil (RBO) oleogel as the representative wax-based oleogel system. RBO was used in this study as it is considered a functional and healthy oil for human consumption. It has several beneficial therapeutic effects such as antioxidant, anti-inflammatory, and hypoglycemic effects. For these reasons, several researchers have employed RBO with various oleogelators to develop oleogels. Recent studies have shown that plant-derived natural waxes act as good gelators for vegetable oils such as RBO [14,15,16]. Some of the widely used plant waxes include candelilla wax, rice bran wax, sunflower wax, beeswax, and carnauba wax. These waxes have been used to prepare different oleogels and oleogel-based food products and delivery systems. Among the plant-derived waxes, SYW is a low-cost component derived from soybean oil. It contains a high percentage of stearic acid (C18:0), which is known to reduce low-density lipoprotein (LDL)-cholesterol. It has also been used in certain food-based applications. For instance, SYW has been utilized to create a superhydrophobic coating that may be applied to packing materials to eliminate liquid food residues without negatively impacting the taste of the contents [17]. However, to the best of our knowledge, SYW is yet to be explored as an oleogelator. The preparation of the optimized RBO/SYW oleogel was followed by adding different emulsifiers. In our study, we used stearic acid (SAC; anionic solid emulsifier), sunflower lecithin (SFL; non-ionic liquid emulsifier), and sorbitan monooleate (SPAN80; non-ionic liquid emulsifier) to study their effects on the prepared oleogels. We hypothesize that the addition of the stated emulsifiers might affect the crystalline network of the produced oleogels, thereby altering the mechanical, thermal, and nutraceutical release properties. The microstructure, color characteristics, mechanical, crystallization kinetics, and X-Ray diffraction of the resulting oleogels were studied to support our notion. The release of curcumin, a hydrophobic nutraceutical ingredient, from these formulations was also investigated. Curcumin-loaded food products have been reported to improve food security by reducing the number of food-borne pathogens.

## 2. Results and Discussion

### 2.1. Visual Appearance of Oleogels

**Figure 1:**
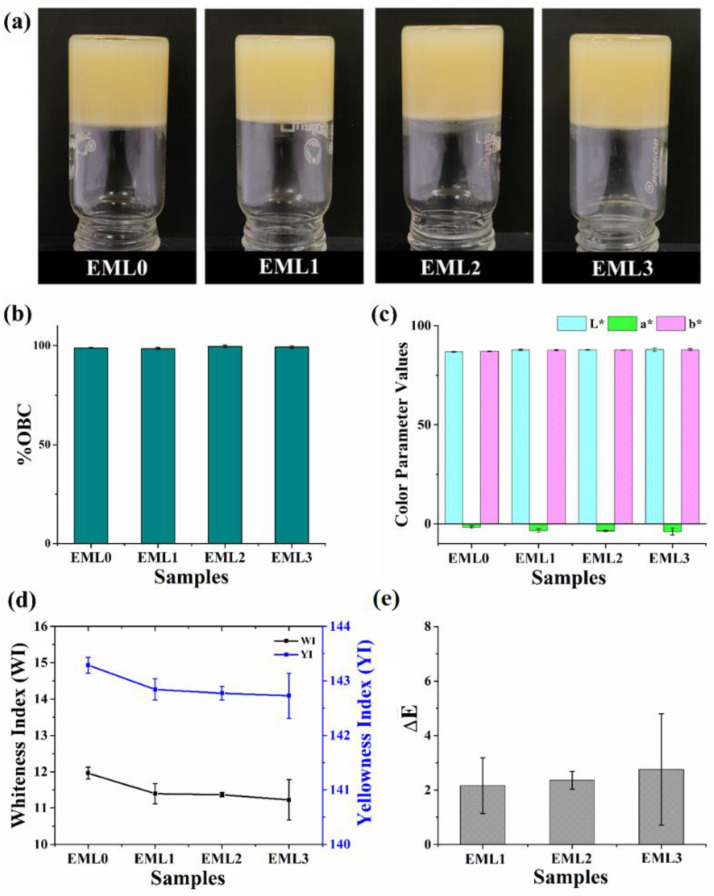
(**a**) Pictograph of prepared oleogels, (**b**) oil binding capacity, (**c**) L*, a*, and b* values, (**d**) whiteness index (WI) and yellowness index (YI), and (**e**) ΔE. The values in the graph are denoted as the average ± SD.

The oleogels containing different emulsifiers are represented in Figure 1a. Following the preparation of the oleogels, glass vials containing oleogels were turned upside down (vial inversion test), and the flow of the samples was observed. SYW prevented the samples from flowing to the bottom of the vial, indicating that it successfully structured the RBO. The color of the prepared oleogels was different from their individual components. Pristine SYW is off-white in appearance, and RBO is brownish yellow in color. RBO produced pale-yellow-colored oleogels that were opaque. The darker hue of RBO and the pale-yellow color of the oleogels are both attributable to the natural colorants (such as anthocyanins, carotenoids, and proanthocyanidins) and phospholipids found in crude vegetable oils [18]. However, adding different emulsifiers did not affect the overall visual perception of the samples as compared to the control sample (EML0). The binding efficiency of the oleogels to the liquid oil was also evaluated. All samples had an OBC rate that was more than 98% (Figure 1a).

### 2.2. Colorimetric Analysis

Color was characterized in the colorimetric study by measuring the reflectance of freshly prepared oleogels using the CIE L*, a*, and b* values. This analysis plays a significant role in evaluating the visual characteristics and probable consumer approval of new food products [19]. The color parameter values were tabulated in Table 1. The L* values of the oleogel samples were in the range of 86–88. Such values indicate that the prepared oleogels were luminous, and the addition of emulsifiers did not compromise the color perception. The a* values were negative and were in the range of −5 to −2 for all oleogels, indicating a higher proportion of green hue. It can be hypothesized that the presence of RBO in oleogels is responsible for their characteristic green hue. The refined RBO contains trace amounts of chlorophyll, which may explain why the oleogels have a green hue [20]. There was a two-point spread between the b* values of oleogels that varied between 86 and 89. The positive values of b* suggest that the oleogel samples had a yellow tone, which is majorly due to the dark yellow color of the RBO [21].

Whiteness, measured by the whiteness index (WI), is quantified by the quantity of visible light a given sample reflects. WI can be correlated to the quality and consumers’ acceptance of food products [22]. WI values for the oleogel samples were in the 11–12 range, with a value of 11.96 ± 0.17 for the control. None of the test samples lost substantial whiteness after being treated with the emulsifiers. To assess the level of yellowness present in our samples, we calculated the yellowness index (YI). An increase or decrease in the YI value emphasizes the white-to-yellow gradient. We observed that there was little to no difference between the YI values of the test samples and EML0. In Figure 1d, the YI values for the oleogel samples were between 142 and 144, which is on the high side. High levels of RBO may account for the characteristic yellow color of oleogels. The WI and YI values suggested that the oleogels were predominantly yellowish. In addition, the ΔE value was established concerning a particular standard. In this instance, we choose to utilize EML0 as the reference. There was no discernible color difference between the test samples and the control (*p* > 0.05) since the ΔE values for the emulsifier-containing oleogels were below 3 [23]. There was also not much difference between the unmodified and modified samples after adding the emulsifier. In general, the color characteristics of the emulsifier-added oleogels were unaffected by the use of emulsifiers. The presence of emulsifiers in the oleogels at such low quantities explains the observed behavior.

### 2.3. Microscopic Analysis

**Figure 2:**
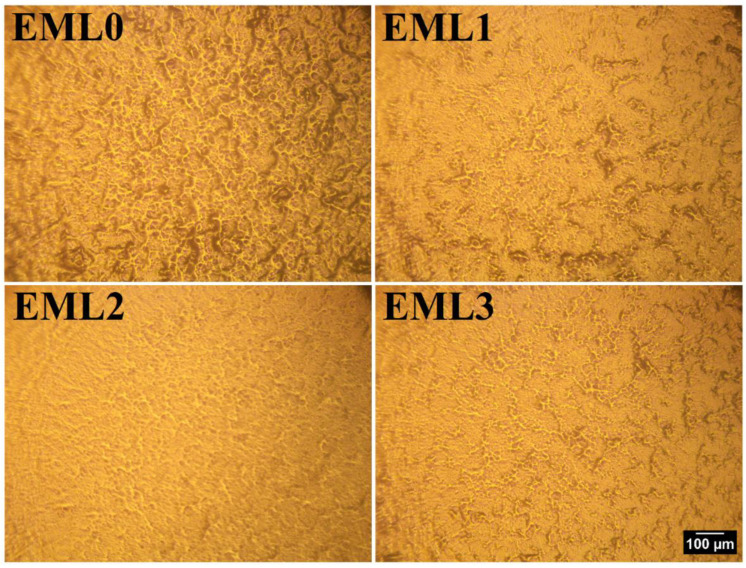
Surface topology of oleogel samples using metallurgical microscopy at 10× magnification. Scale: 100 µm.

The surface topologies of the emulsifier-added oleogels are shown in Figure 2. As seen from the figure, the inclusion of emulsifiers changed the surface topologies of the oleogels. In the control sample (EML0), well-connected dense fibrous structures and some dark regions were observed. Compared to EML0, the density of the fibrous structure and the dark regions decreased in EML1. Moreover, the fibrous structures were not well connected, and to some extent, the fibrous structures did not participate in branching. Thus, a more smooth surface appeared in EML1, which can be attributed to the presence of SAC, which forms smooth and opaque oleogels [24]. EML2 showed a much smoother surface than EML1, with minimum dark patches; however, the fibrous structures were visible to a certain extent. Lastly, in EML3, small but well-connected fibrous structures can be seen from the surface topology. However, the dark patches were negligible compared to the control sample. Therefore, it can be concluded from the surface topography that the addition of emulsifiers changed the surface topologies of the oleogels.

**Figure 3:**
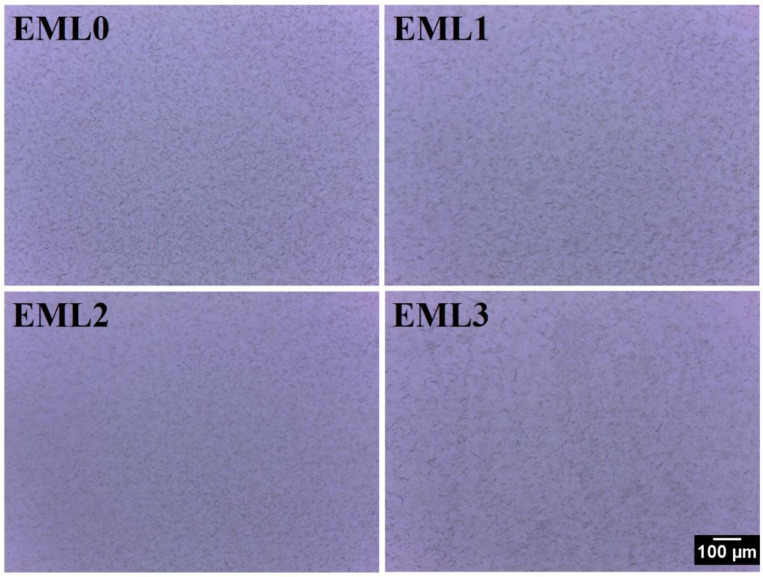
Bright-field micrographs of the oleogel samples at 10× magnification. Scale: 100 µm.

Analysis of the internal structure of the oleogels was conducted by inspecting the samples using bright-field and polarized light microscopes. Figure 3 represents the bright-field micrographs. The bright-field micrograph of EML0 showed the presence of compactly packed small wax crystals. Some of these crystals were in the form of needle-shape, and some were small globular structures. However, both morphologies were present in similar densities. Such morphology is in accordance with the fact that some plant-derived waxes show needle-like crystal morphology (e.g., rice bran wax and sunflower wax), and some show granular/globular crystal structure (e.g., candelilla wax). In EML1, the addition of SAC did not change the crystal morphologies; however, it dispersed the distribution of wax crystals compared to EML0. Previously, studies had reported that SAC formed a similar shape of crystals when stearic acid was solely used as an oleogelator to structure sunflower oil [25]. The development of oleogels is aided by crystals of this shape because they may entrap more liquid oil. The addition of SFL decreased the wax crystal size, as observed from the micrograph of EML2. Moreover, there was an increase in the proportion of wax crystals showing grainy morphology instead of needle-shaped crystals. The micrograph of EML3 showed the presence of prominently large and thin needle-shaped crystals along with a few globular patches. We also observed the branching of the needle-shaped crystals in EML3 to some extent. The needle-shaped crystal morphologies can better interact with each other and form a 3D network that can successfully entrap the liquid oil and form stable oleogels. This may help to explain why the prepared oleogels contain such a high amount of OBC, as shown in Figure 1b.

**Figure 4:**
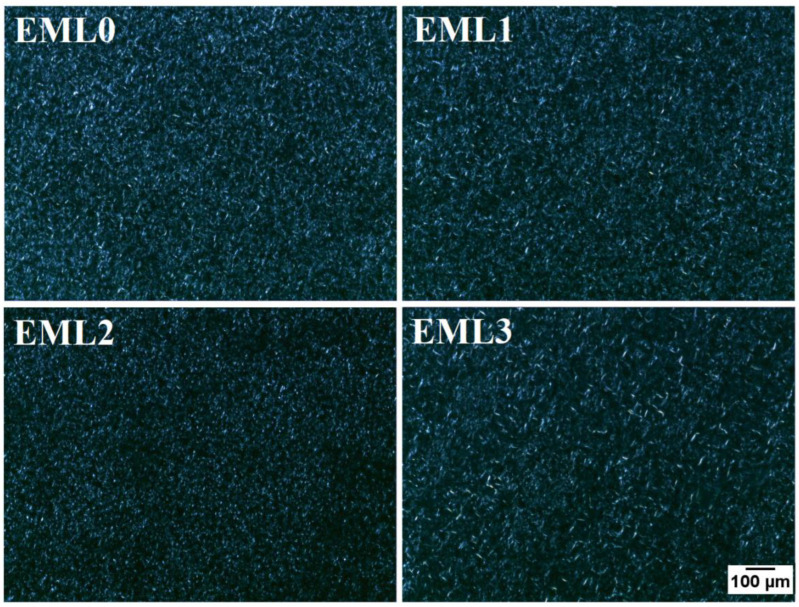
Polarized light micrographs of the oleogel samples at 10× magnification. Scale: 100 µm.

The oleogels were further visualized under the polarized light microscope (PLM) to obtain better contrast images of the internal microstructures of the oleogels (Figure 4). The wax crystals appeared as bright structures under polarized light, and the black background represents the optically isotropic amorphous region of liquid RBO. These findings are consistent with research conducted by Okuro and co-authors (2018). They investigated the gelation of fruit wax using sunflower oil treated with lecithin as a crystal modifier. Under polarized light, sunflower oil appeared as a dark background, with fruit wax as bright white crystals. Natural plant-derived waxes usually contain long-chain hydrocarbons belonging to fatty acids, fatty alcohols, and fatty acid esters. The presence of such molecules, especially esters, triggers intermolecular interactions such as van der Waals and hydrophobic interactions. Such interactions help form distinctive needle-shaped wax crystals and govern the physical characteristics of the oleogels [26].

EML0 had bright needle-shaped and small globular wax crystals, which seemed homogenously distributed throughout the sample. EML1 showed similar crystal morphology, but the crystal distribution density decreased compared to the control. Additionally, the thickness of needle-shaped crystals increased in EML1. Lecithin-containing oleogel sample, EML2, had small grainy morphology of the wax crystals, which seemed well distributed throughout. Hydrophobic fatty acid tails are esterified to a glycerol backbone in lecithin together with phospholipids that have a hydrophilic charged headgroup. The presence of such chemical groups helps lecithin molecules self-assemble, creating a synergistic effect within the oleogel [27]. In a previous study, the authors reported the appearance of globular wax crystals when SFL and SYL were used to determine the structural variations of candelilla wax and RBO-based oleogels [28]. Such microarchitecture was attributed to the interaction between minor components and major alkanes in wax molecules. EML3 showed large wax crystals, giving an appearance of a mesh-like structure. The formation of such microarchitecture can be related to the high ester content in SPAN80 and SYW. Uslu et al. (2021) reported that the addition of SPAN80 into sunflower oil and glycerol monostearate oleogels increased the size of the lipid crystals and formed large aggregates [29]. These emulsifiers act as wax crystal modifiers whose structure is comparable to the participating wax molecules. Reports suggest that these crystal modifiers usually contain one or more non-polar hydrocarbon chains and a polar portion. Such functional groups can co-crystallize along with the wax molecules, hence occupying the position of wax crystals on the crystal lattice. Such molecular arrangements can create steric hindrance that interferes with the growth and aggregation of wax crystals in the 3D network [30]. This can explain why the crystalline structure of fat crystals changes and how they are spread out in the microstructure of oleogels with an emulsifier added. However, the changes made by these methods have led to a higher space-filling network, which makes the SYW/RBO-based oleogels more stable.

### 2.4. FTIR Analysis

**Figure 5:**
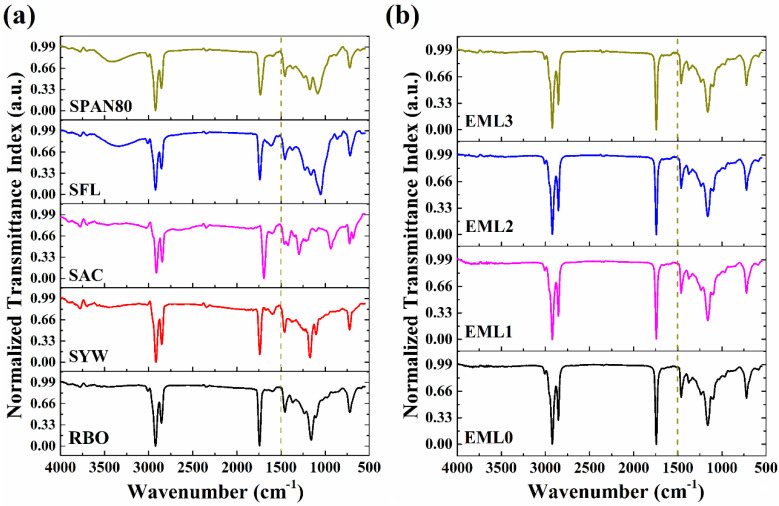
FTIR spectrum of (**a**) raw materials and (**b**) oleogel samples.

Figure 5 shows the typical ATR-FTIR spectra of raw materials and the emulsifier-added oleogels. The spectral information was recorded in the wavenumber ranging from 500 to 4000 cm^−1^. We divided the spectrum into two parts: (i) the region between 4000 and 1500 cm^−1^, called the diagnostic region of the functional groups present in the compound, and (ii) the region below 1500 cm^−1^, called the fingerprint region [31].

The FTIR spectrum of the RBO (Figure 5a) revealed the presence of various peaks at wavenumber 3009, 2922, 2856, 1744, 1457, and 720 cm^−1^. The symmetric stretching vibrations of the cis-olefinic double bonds are responsible for the small spike seen at 3009 cm^−1^. This peak demonstrated the presence of the linoleic and linolenic acyl groups in RBO [32]. The sharp peaks at 2922 and 2856 cm^−1^ are mainly due to the triglycerides comprising 83–86% of total neutral lipids in RBO. The major peak at wavenumber 2922 cm^−1^ can be associated with the asymmetric stretching vibration of the methylene groups of triglycerides [33]. The peak at 2856 cm^−1^ represents the symmetric C-H stretching vibration of aliphatic CH_2_ functional groups in triglycerides. C=O stretching of the carbonyl group in the esters of triglycerides can account for the significant rise at 1744 cm^−1^ [34]. In the fingerprint region of RBO, a sharp peak of medium intensity was observed at ~1457 cm^−1^, which correlates to the bending vibrations of -CH_2_ [35]. Lastly, a prominent peak was observed at ~720 cm^−1^, which can be attributed to the superposition of the CH_2_ rocking vibration and the out-of-plane vibration of the cis-disubstituted olefins (-HC=CH-) [36]. The peaks obtained in the FTIR spectra of RBO were also observed for the spectra of SYW (Figure 5a). However, some additional peaks were observed for SYW. A few small peaks were observed at around 3785 cm^−1^ which can be attributed to the hydroxyl group stretching vibrations. Such peaks could have arisen due to the presence of natural waxes comprising long-chain alcohols, sterols, and sterol esters [37]. The small peak obtained at 1600^−1^ can be assigned to the stretching vibrations of C=C, which confirms the presence of unsaturation in SYW. The C-O stretching of the alcohol groups in SYW accounts for a shoulder peak at 1100 cm^−1^.

The FTIR spectra obtained for SFL and SPAN80 showed the presence of similar peaks (Figure 5a). The absorption band at 3500 cm^−1^ relates to the stretching vibrations of O-H of the lipid structure. The peaks at 2922 and 2854 cm^−1^ can be ascribed to the stretching vibration of aliphatic hydrocarbons such as -CH_2_- and -CH_3_ [38]. The C=O stretching was responsible for the sharp peak at 1735 [39]. The peak obtained at 715 cm^−1^ with a medium intensity was due to the in-plane asymmetric deformation of CH_2_. The absorption spectrum of SAC showed peaks at 2911, 2846, 1693, 1295, 934, and 677 cm^−1^ (Figure 5a). The asymmetric and symmetric stretching vibrations in the aliphatic CH_2_ band are responsible for the adsorption peaks in the high-frequency region that occur at approximately 2911 and 2846 cm^−1^, respectively [40]. The sharp peak at 1693 cm^−1^ can be attributed to the characteristic stretching vibrations of the carbonyl (C=O) groups [41]. In the low-frequency region, the SAC spectrum showed several medium-intensity peaks. One of these peaks, seen at 1295 cm^−1^, represents the in-plane bending vibration of the -OH group in the SAC [42]. Further in the spectrum, a broad and medium peak was observed at 934 cm^−1^, which can be related to the out-of-plane bending vibration of the hydroxyl group (CO-OH). The existence of polymorph A of SAC is indicated by the presence of a peak at 677 cm^−1^, which is associated with a distortion of the O-C=O bond angle [43]. Additionally, the presence of peaks at 1461 and 722 cm^−1^ also corresponds to the presence of polymorphs (B and C) of SAC.

Figure 5b represents the FTIR spectrum of the control and emulsifier-containing oleogel samples. In the control sample (EML0), peaks were obtained at the wavenumbers 3009, 2922, 2852, 1744, 1463, 1373, 1163, 1095, and 720 cm^−1^. As EML0 is composed only of RBO and SYW, the peaks obtained in EML0 are similar to those obtained in RBO and SYW. It was further observed that the spectrum of the emulsifier-containing samples was similar to that of EML0. This similarity might be ascribed to the higher RBO and SYW concentrations in the test samples compared to the emulsifiers. In other words, the RBO and SYW concentrations outweighed the emulsifier concentrations. The peak for intermolecular hydrogen bonding was absent in the region between 3700 and 3100 cm^−1^ of the test samples. Thus, the oleogel samples had no/negligible intermolecular hydrogen bonding among the constituents. Waxes are the low molecular weight oleogelators known to yield fibrous networks by self-assembling the small molecules. The self-assembly process mainly involves the non-covalent interactions (e.g., van der Waals forces, hydrophobic interactions, and π–π stacking) among the gelator molecules, which results in the immobilization of many solvent molecules. Therefore we can hypothesize that the formation of stable formulations can be attributed mainly to the non-covalent interactions [44]. Furthermore, a small peak was seen in all of the samples at 3009 cm^−1^. This peak is caused by the symmetric stretching vibration of alkene double bonds, which suggests that all of the samples are unsaturated [45].

### 2.5. Mechanical Analysis

**Figure 6:**
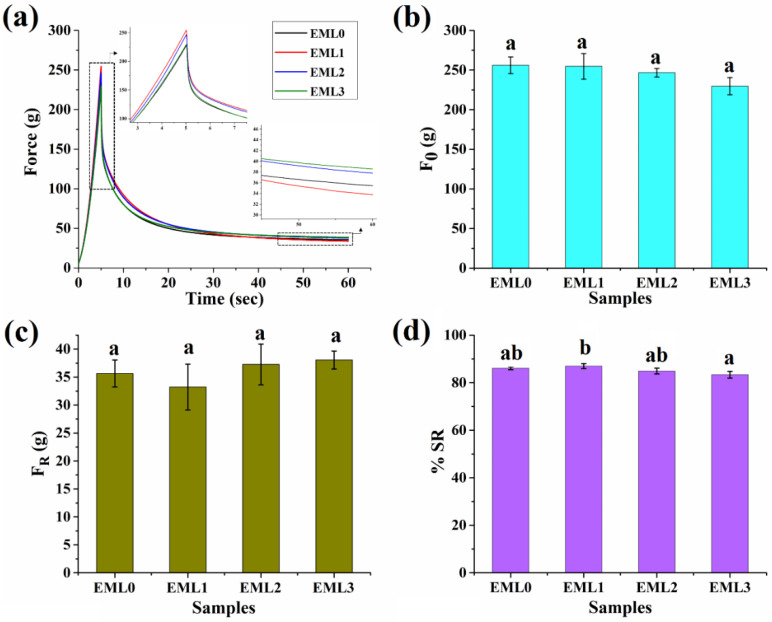
Mechanical properties of oleogel samples. (**a**) Stress relaxation profile, (**b**) F0 values, (**c**) FR values, and s(**d**) %SR values. Different letters represent significantly different values (*p* < 0.05).

The purpose of the mechanical study was to understand the changes that occurred in the characteristics of the oleogel due to the incorporation of emulsifiers at high degrees of deformation. As a result, the stress relaxation (SR) investigation was carried out to investigate the viscoelastic properties of the oleogels following deformation. Stress relaxation refers to a time-dependent reduction in stress under persistent strain. After a given amount of deformation, the internal matrix of a material relaxes along molecular chains as the stress is dissipated exponentially. If the distortion and the resulting gentle springing back together happen instantly, we call the phenomena “elastic.” Gels, on the other hand, have a time-dependent spring-back action, and the stress decays with time in a manner analogous to molecular relaxation. During the compression phase of the stress relaxation study, the polymer chains are stretched, which causes a change in their physicochemical characteristics, which in turn controls the relaxation process. Importantly, the physical contact between the polymer and its side chains is one of the features that changes throughout the relaxing process [46]. The typical SR curves of the finished oleogels are shown in Figure 6a. In addition to a high % OBC, the great mechanical strength of the oleogels provides further evidence that the gels are very stable. The firmness of the oleogel samples is shown by their maximal force (F0 values) under strained conditions (Figure 6b). For comparison, the firmness value of the EML0 was 255.95 ± 10.55 g. There was no significant difference between F0 values of EML0 and emulsifier-containing oleogels. After 60 s of continual strained circumstances, the force values reduced exponentially to a minimum constant force value (F_R_) during the relaxation period. This decrease in force values may be ascribed to either network chain relaxation or rupture of the network established by the gelator molecules (herein, wax crystals). The residual force, or F_R_, denotes the elastic component of the oleogels. The F_R_ values of the emulsifier-added samples were comparable to the control (EML0).

Figure 6d represents the %SR profile of the oleogel samples. SR provides information on how viscoelastic materials release stress under constant strain conditions as a function of time. Ideal fluids have a %SR of 100%, while ideal elastic materials have a %SR of 0% [47]. If the %SR of a polymer is high, then it means the network of that polymer may relax or be readily disturbed by force. Having a %SR in the 83–87% range, oleogels are supported as viscoelastic liquids. The %SR for the control group was 86.07 ± 0.59. Noticeably, the %SR of EML3 was significantly lower than EML1. Such observation can be reasoned to the formation of a stronger and a rigid 3D network in the presence of SPAN80. Further, the strong gel network obstructs the movement of the oil droplets resulting in a stiffer solid formulation that exhibits lesser relaxation behavior.

### 2.6. Crystallization Kinetics

The formation of oleogels is carried out by dissolving the gelator molecules in heated oil, and then the system is cooled to a temperature below the solubility limit, usually referred to as the Krafft temperature [48]. Following that, a process of rearrangement of gelator molecules begins, eventually leading to nucleation. The crystalline structures that emerge from newly formed nuclei result from a succession of dimensionality patterns and crystal growth geometries. Once nucleation occurs in oleogels, the gelator molecule initiates a phase of primary crystallization and structural growth. This mechanism is similar to the crystallization of fat molecules. Primary crystallization occurs rapidly; during this period, the nuclei grow outward, producing smaller and less perfect crystals. The primary crystallization is then followed by a secondary crystallization process that occurs at a slow pace. During this process, the crystallites might become thicker, and/or new crystals might form, thus reducing the amorphous region. Following secondary crystallization, an equilibrium phase is obtained where no more new crystals are formed.

**Figure 7:**
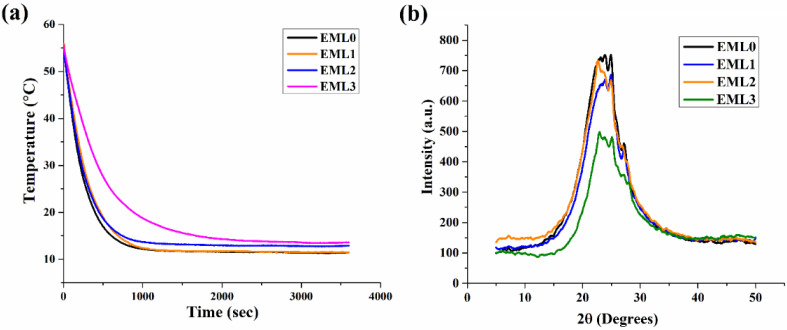
(**a**) Crystallization kinetics profile of the oleogel samples and (**b**) XRD diffractogram profile of the oleogel samples.

The nature of the crystallization process in the prepared oleogels can be determined by monitoring it as a function of time. Figure 7a represents the profile of crystallization kinetics for oleogel samples. Initially, a steep decrease in the temperature (55–35 °C) was observed in all the samples. This marks the initiation of the nucleation phase, in which the primary crystallization takes place by forming nuclei in the amorphous region. The crystal nuclei were probably undetected in the micrographs due to the limited resolution of PLM. However, small grain-like crystals were clearly observed in the micrographs. The nucleation phase was followed by an intermediate stage showing a slow temperature reduction between 35 and 20 °C. In this temperature range, a curve in the kinetics profile was observed, which specifies the start of secondary crystallization. The secondary crystallization in the control sample, EML0, was observed at around ~294 s. The onset of secondary crystallization has been tabulated in Table 2. The secondary crystallization in EML0, EML1, and EML2 were similarly valued. Interestingly, the onset of secondary crystallization in EML3 was the slowest among all the oleogels. The slowest crystallization process might have contributed to the formation of large and better wax crystals. This is in accordance with the PLM micrograph of EML3, which showed the presence of large needle-shaped crystals.

### 2.7. XRD Analysis

XRD is a technique that can provide in-depth knowledge of the packing arrangement and crystallite size of the wax crystals in the non-polar oil phase. It also provides information on the polymorphic forms of the lipid structures that might contribute to the structural properties of the oleogels upon the addition of emulsifiers. The wide-angle or short-spacing XRD diffractograms (θ > 20°) of the oleogel samples are shown in Figure 7b. This region displays the lateral packing order of the hydrocarbon or the fatty acid chains in the 3D network of the oleogels. Therefore, the lateral packing was calculated using Brag’s Law and represented using Brag’s distance (d-spacing). Generally, the basic lipid polymorphs include α, β, and β′, where the fatty acid chains are packed where the chains are organized in hexagonal, triclinic, and orthorhombic structures, respectively. However, the presence of unsaturated fatty acids in RBO (linoleic and oleic acid) and their polymorphs might further contribute to the peaks obtained in the wide-angle region of XRD diffractograms.

Polymorphs of SYW and unsaturated fatty acids of RBO contributed to the generation of diffractogram peaks in the control sample (EML0). Important diffraction peaks were seen at 23.17° 2, 24° 2, 24.98° 2, and 27.05° 2θ for EML0. Analysis of the d-spacing values indicated that there may have been many polymorphic forms of wax crystals present in the prepared sample. The magnitudes of these peaks have been augmented or diminished, depending on the emulsifier used (Figure 7b). The primary peaks of EML0 and EML1 were in similar locations. However, the peak intensity was lowered in EML1, and it can be reasoned that EML1 had a smaller crystallite size than the control sample. EML2 also had a similar diffractogram peak as that of EML0. EML3 showed a broad peak of lower intensity, indicating the presence of large crystals. This was also evident in PLM micrographs, which showed the presence of long needle-shaped crystals in the micrograph of EML3.

**Figure 8:**
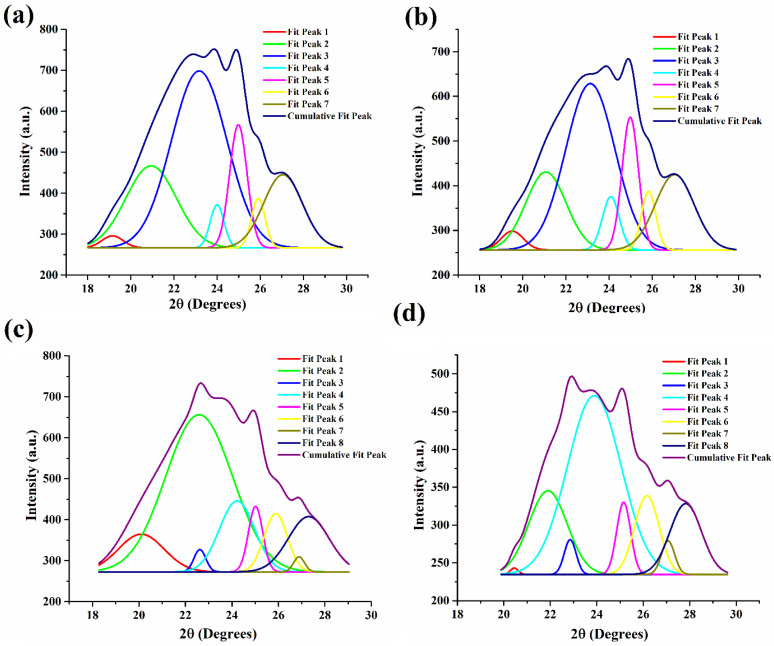
Deconvoluted XRD peaks. (**a**) EML0, (**b**) EML1, (**c**) EML2, and (**d**) EML3.

XRD profiles were deconvoluted using Origin 9 Pro and Gauss peak fitting technique to compute average d-spacing, crystallite size, and lattice strain (Figure 8 and Table 2). We observed that the average d-spacing values for EML0 (4.43 Å) and EML1 (4.41 Å) are identical. Furthermore, the average d-spacing values for EML2 (4.29 Å) and EML3 (4.28 Å) were quite close. Overall, it was observed that the average d-spacing for EML3 was the lowest among all the oleogel samples. Such observation indicates that wax crystals present in EML3 must have been arranged in close vicinity, forming a stable structure. This was evident from the SR study, where it was observed that EML3 had the lowest average %SR value, which was significantly lower than EML1 (*p* < 0.05). A sample with a lower %SR is related to the higher elastic component, a marker of the inherent stability of the sample.

In the control sample (EML0), we discovered that the average crystallite size was 8.06 nm (Figure 8a; Table 2). However, in EML1 (Figure 8b), the crystallite size was found to have shrunk ever-so-slightly (7.88 nm). The incorporation of SAC, which may have inhibited the development of bigger wax crystallites, can be linked to this decrease. Interestingly, EML2 and EML3 exhibited bigger crystallites than the control sample (Figure 8b,c). This indicates that the presence of SFL and SPAN80 facilitated the growth of larger crystallites. The largest crystallites were produced in EML3, which contained SPAN80, compared to the other oleogel samples (Figure 8d). A slower crystallization rate often results in the formation of bigger crystallites. The crystallization kinetics study showed that EML3 crystallized the slowest, yielding the biggest crystallites (average size of 11.54 nm). Lastly, the findings verify, by mechanical analysis, that the bigger crystallites of EML3 have contributed to its stable and rigid network. All samples exhibited little lattice strain, suggesting that the wax crystallites used to make the oleogels had few imperfections.

### 2.8. Drug Release Study

**Figure 9:**
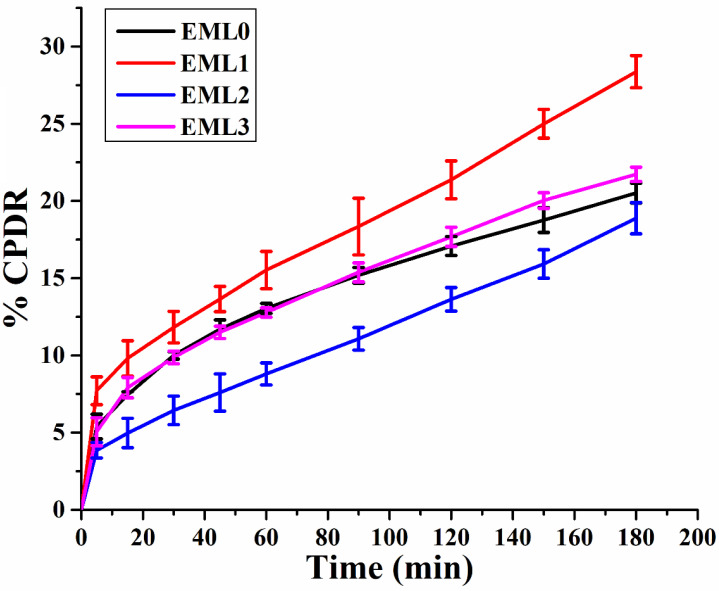
Drug release study of oleogels showing cumulative percent drug release (CPDR) profiles (average of 3 replicates).

It has been shown that oleogels are an effective matrix for delivering bioactive molecules since they prevent the bioactive compounds from deteriorating due to oxidation and regulate their release. Many oleogel-based delivery methods employ the bioactive component curcumin (CUR) as a model nutraceutical due to its beneficial effects on human health. CUR is not very bioavailable or bioaccessible due to its rapid metabolism and insolubility in water. We can learn more about the potential of the prepared oleogels as a controlled-release delivery system by analyzing the release kinetics of CUR. Accordingly, CPDR values for CUR were calculated from the oleogels, and the resulting release patterns are shown in Figure 9. It can be observed that the emulsifier-added oleogels had tailored CPDR profiles of CUR compared to the control (EML0). EML0 had a CPDR of 20.51%. The CPDR values of emulsifier-added oleogels varied in the order of EML1 < EML3 < EML2 at the end of the experiment. Oleogel samples showed a controlled release of CUR, wherein a maximum CPDR (28.37%) was observed in EML1, and a minimum CPDR (18.88%) was observed in EML2. In the current study, the release study was performed at pH 6.8; however, we intend to perform the release study at gastric and basic pHs in the future to have an understanding of the pH-dependent release behavior of CUR from the oleogels.

## 3. Conclusions

In conclusion, this research set out to determine how adding various emulsifiers (namely, SAC, SFL, and SPAN80) might affect the SYW/RBO-based oleogels. The physiochemical behavior of the oleogel samples was assessed using colorimetry, microscopy, FTIR, mechanical, crystallization kinetics, X-ray diffraction analyses, and a drug release study. Prepared oleogels appeared light yellow, and the addition of various emulsifiers did not alter their overall look. Despite varying emulsifier additions, all SFW oleogels showed OBCs over 98%. Sample surface topographies indicated that the emulsifiers had improved the surface smoothness of the oleogels over the control sample. It was clear in bright-field and PLM micrographs that the wax crystals in the oleogels were present in both grain and needle-shaped architectures. Wax crystals in EML2 were more like grains, whereas those in EML3 were more like long needles. FTIR analysis of the oleogel samples confirmed that they, too, included a wide variety of functional groups, much like the original ingredients. However, unlike SYW and other emulsifiers, the oleogel samples showed no hydrogen-bonding signal. Thus, we reasoned that non-covalent interactions must have played a significant role in developing oleogel samples. All oleogel samples showed signs of unsaturation, according to FTIR analysis. Insight into the mechanical characteristics of the oleogel samples was gained by a stress relaxation analysis. Stress relaxation studies demonstrated that emulsifiers did not affect the oleogels’ firmness (F0) or elastic component (FR). The %SR readings did, however, change significantly in some cases. When compared to the other oleogels containing emulsifiers, EML3 had superior inherent stability. According to microscopic examination, EML3 crystallized at the slowest rate, leading to the largest crystallites. XRD diffractograms also showed that the crystal molecules of EML3 are packed very tightly. These results confirmed that the addition of SPAN80, which encouraged the formation of large crystals, was responsible for EML3′s prolonged crystallization rate. Additionally, the developed emulsifier-containing oleogels considerably affected the release of curcumin from the oleogel matrices. These results suggest that the developed oleogels have potential applications in the food and pharmaceutical industries due to their ability to regulate the release of bioactive components.

## 4. Materials and Methods

### 4.1. Materials

Edible-grade RBO (Fortune Rice Bran Health, Adani Enterprises Ltd., Ahmedabad, India) used in the experiment was purchased from the local shop. Triple-filtered SYW was acquired from Tattvalogy (Mekasa Products Pvt. Ltd., New Delhi, India). SFL and SYL were obtained from Urban Platter, Ltd., Maharashtra, India. SAC (C18H36O2; Octadecanoic acid) and SAL (C18H38O; 1-Octadecanol) were procured from Himedia Laboratories Pvt. Ltd. Mumbai, India. SPAN80 (C24H44O6) was purchased from Loba Chemie Pvt. Ltd., Mumbai, India.

### 4.2. Preparation of Oleogels

The critical gelation concentration (CGC) of SYW for gelling RBO was initially determined by varying SYW concentrations from 1% to 13% *w*/*w*. The CGC was found to be 13% *w*/*w*. For improved stability of the oleogels, we used 15% *w*/*w* of SYW for our experiment. For the control sample (EML0), the required amount of SYW and RBO were weighed accurately and placed in a beaker. The beaker was covered with aluminum foil and kept in a water bath at 65 °C for 15 min. Then, the mixture was stirred (300 rpm) for 20 min on a magnetic stirrer to form a homogenous solution of SYW and RBO. The obtained homogenous clear solution was transferred into a sample box. The sample box was then kept in a thermal incubator (25 °C) for one hour without any shear effect so that oleogelation would take place. The gel formation was validated using the inverted tube approach, which involved observing the material flow under gravity.

On the other hand, five different stock solutions of 0.1% (*w*/*w*) each of the emulsifiers SAC, SFL, and SPAN80 were prepared in RBO. The emulsifier stock solution was added to make a series of formulations such that the final concentration of emulsifier in the oleogels would be 5 mg (Table 3). The emulsifier-added oleogels were then prepared with a similar method as explained above.

The nutraceutical-loaded oleogel samples were prepared by dissolving the CUR in the molten oleogels at 5mg/g (*w*/*w*). These molten samples were then subjected to similar conditions explained above to form oleogels. Since the CUR-loaded oleogels were prepared by the direct dispersion method, the encapsulation efficiency of CUR was 100%.

### 4.3. Characterization

#### 4.3.1. Oil Binding Capacity (OBC)

The OBC of the oleogels was determined by the centrifugation method [11]. An empty 2 mL Eppendorf was initially weighed (A), and ~1 g of the molten samples were poured into the Eppendorf. The sample-containing Eppendorf was weighed (B) and was allowed to solidify in the thermal incubator at 25 °C for 24 h. The OBC was measured by centrifuging (Remi C-24 BL refrigerated centrifuge) the Eppendorf at 10,000 rpm for 15 min at room temperature (25 °C). Following centrifugation, the released oil was decanted, and the weight of the Eppendorf was measured again (C). The % of released oil and % OBC of each sample were calculated in triplicate using the following equations:

(1) $$\%\;\mathrm{Released}\;\mathrm{Oil}=\left[\frac{\left(B-A\right)-\left(C-A\right)}{\left(B-A\right)}\right]\times 100$$

where A is the weight of empty Eppendorf, B is the weight of the oleogel containing Eppendorf, and C is the weight of the Eppendorf after centrifugation and removal of released oil.

(2) % OBC=100−% Released Oil

where % OBC is the percentage of oil binding capacity.

#### 4.3.2. Colorimetry

An in-house-built colorimeter was used to assess the color of the formulated oleogels. Initially, the instrument sensor was calibrated using black and white reference cards to ensure measurement accuracy and repeatability [49]. This analysis was performed by placing the molten oleogels in 35 mm Petri dishes and incubating them at 25 °C for gel formation. For each sample, the CEI Lab color parameters, including L*, a*, and b*, were measured on several points of the Petri plates filled with oleogels. L* signifies the darkness to lightness parameter; a* denotes greenness to redness, and b* indicates blueness to yellowness. The values of the parameters mentioned above were further used to calculate the chromatic parameters, including the whiteness index (WI), yellowness index (YI), and absolute color difference (ΔE). The following formulae were used for calculating the color indexes [50]:

(3) $$\mathrm{WI}=100-\sqrt{{\left(100-L^{*}\right)}^{2}+{\left(a^{*}\right)}^{2}+{\left(b^{*}\right)}^{2}}$$

(4) $$\mathrm{YI}=142.86\frac{b^{*}}{L^{*}},$$

(5) $$\Delta E=\sqrt{{\left(L_{C}^{*}-L_{z}^{*}\right)}^{2}+{\left(a_{C}^{*}-a_{z}^{*}\right)}^{2}+{\left(b_{C}^{*}-b_{z}^{*}\right)}^{2}},$$

#### 4.3.3. Microscopy

##### Surface Topology

A metallurgical microscope (AMscope, ME580TA, Irvine, CA, USA) and an external eyepiece lens camera (MU1603, AMscope, Irvine, CA, USA) were used to visualize the surface of the prepared oleogels under a magnification of 10×. Before the experiment, the molten oleogels were transferred to 35 mm Petri dishes and placed in a thermal cabinet (25 °C) for 24 h.

##### Crystal Morphology

The microstructure showing the crystal morphology of the prepared oleogels with different emulsifiers was visualized at room temperature using an upright bright-field compound microscope (Leica Microsystems, model: DM750, GmbH, Wetzlar, Germany), and the images were captured with ICC 50-HD camera. The microscope was coupled with an in-house polarizer to visualize the micrographs in polarized mode.

#### 4.3.4. Fourier Transform Infrared (FTIR)

The IR-absorption spectra of the raw materials and the prepared oleogels were obtained using an FTIR spectrophotometer (Alpha-E; Bruker, Bremen, Germany). The instrument was coupled with an attenuated total reflectance (ATR) Zinc selenide (ZnSe) crystal. The samples and raw materials were scanned in ATR mode with wavenumber ranging from 4000–500 cm^−1^, each with 25 scans at a spectral resolution of 4 cm^−1^.

#### 4.3.5. Mechanical Study

The viscoelastic properties of the oleogels were investigated by stress relaxation (SR) studies. The experiment was carried out using a texture analyzer (TA-HD plus instrument, Stable Microsystems, Godalming, Surrey, UK). The molten oleogels (~40 g) were poured into 100 mL polypropylene beakers and kept at 25 °C to solidify for 24 h. The instrument was equipped with a 45° acrylic male cone for the SR study. The measurements were taken by the compression mode in which the conical probe penetrated the solid oleogels to a distance of 5 mm (1 mm/s) after a trigger force of 5 g. The test was conducted at a constant strain of 5.0 mm at a rate of 1 mm/s. Then, the strain was maintained for a period of 60 s. Lastly, the probe was moved back to its initial position. The changes in the force values were recorded. %SR of the oleogels was calculated using the force values in Equation (6).

(6) $$\%\mathrm{SR}=\frac{F_{0}-F_{R}}{F_{0}}\times 100$$

where F_0_ is the maximum force, and F_R_ is the residual force.

#### 4.3.6. Crystallization Kinetics

An in-lab-developed temperature sensing device was used to study the crystallization kinetics of the prepared oleogels. Molten oleogel samples were transferred into the 15 mL culture bottles and heated at 65 °C in a water bath. Then, the temperature sensors (Model: DS18b20, Digital Temperature sensor, Kuongshun Electronic, Shenzhen, China), connected to the sensing device, were attached to each of the culture bottles containing molten oleogels and placed into a sample holder. The sample holder was then dipped into a refrigerated water bath (Equibath, Refrigerated Circulating, Equitron Medica Private Limited, Mumbai, India), maintained at 5 °C. The temperature changes were recorded using a Python program. The change in the temperature was recorded for 60 min after 55 °C was achieved.

#### 4.3.7. X-ray Diffraction

X-ray diffractometer (D8 Advance with DAVINCI design, Bruker, Austin, TX, USA) was used to record the XRD patterns of the prepared oleogel samples. The X-ray source employed was Co-Kα radiation, which was run at 35 kV and 25 mA (λ = 1.79 Å). The oleogel samples were examined at a rate of 5° 2θ/min with diffraction angles ranging from 5° to 50° (2θ). Data analysis was accomplished by Philips X’Pert HighScore software (Malvern Panalytical, Malvern, UK). The d-spacing and crystallite size were calculated using Brag’s law (Equation (7)) and the Debye–Scherrer equation (Equation (8)). The lattice strain (ε) was calculated using Equation (9).

(7) λn=2dsinθ,

where λ = 1.79 A°, n = integer value, and θ = diffraction angle.

(8) $$D=\frac{\lambda \;k}{\beta \cos\theta },$$

where k = Scherrer constant, β (radian) = full width at half maxima (FWHM) at 2θ.

(9) $$\varepsilon =\frac{\beta }{4\tan\theta },$$

where ε = lattice strain, β (radian) = FWHM at 2θ, and θ = diffraction angle.

#### 4.3.8. Drug Release Kinetics

The nutraceutical (CUR) release profile from the oleogel samples was evaluated using a tablet dissolution test apparatus (DS 8000, Lab India Instruments Pvt. Ltd., Mumbai, India). The test was conducted in phosphate buffer saline (PBS) with a pH of 6.8 containing 0.25% (*w*/*v*) sodium lauryl sulfate. Amounts of ~1 g of the nutraceutical-loaded oleogel samples were kept in the USP type I basket (sample holder) and inserted in the dissolution vessel containing 500 mL of PBS. The test was conducted for three hours at 37 ± 0.2 °C. The basket was rotated at a constant speed of 100 rpm. Subsequently, 5 mL of aliquots were withdrawn using a syringe at regular intervals (5, 15, 30, 45, 60, 90, 120, 150, and 180 min) and replaced by an equal volume of fresh PBS. The aliquots were then analyzed for the presence of CUR using a UV–visible spectrophotometer (UV-1900i, Shimadzu Corporation, Kyoto, Japan) at 445 nm. The nutraceutical release kinetics from the oleogel samples was expressed as the release percentage with respect to time.

#### 4.3.9. Statistical Investigation

The samples were tested in triplicate, and the results were given as the average ± standard deviation (SD). The IBM SPSS Statistics (Version 20) software program was used for the statistical analysis. The data were analyzed using one-way ANOVA followed by post hoc Tukey HSD comparisons at a 95% confidence level. When *p* < 0.05, the results were considered statistically significant.

## Figures and Tables

**Table 1:** Color parameters of the oleogels. For each column, values having the same letter(s) are similar to each other by the Tukey HSD test.

Sample	Color Parameter Values					
	L*	a*	b*	WI	YI	ΔE
EML0	86.76 ± 0.29 ^a^	−1.58 ± 0.58 ^b^	87.02 ± 0.20 ^c^	11.96 ± 0.17 ^d^	143.29 ± 0.14 ^e^	-
EML1	87.68 ± 0.43 ^a^	−3.44 ± 0.87 ^b^	87.67 ± 0.31 ^c^	11.40 ± 0.28 ^d^	142.84 ± 0.20 ^e^	2.17 ± 1.02 ^f^
EML2	87.76 ± 0.14 ^a^	−3.61 ± 0.29 ^b^	87.71 ± 0.07 ^c^	11.37 ± 0.06 ^d^	142.77 ± 0.12 ^e^	2.36 ± 0.33 ^f^
EML3	87.93 ± 0.87 ^a^	−3.94 ± 1.75 ^b^	87.85 ± 0.61 ^c^	11.23 ± 0.55 ^d^	142.73 ± 0.41 ^e^	2.76 ± 2.05 ^f^

**Table 2:** XRD parameters obtained from the deconvoluted peaks.

Sample	Peaks	Peak Position (°2θ)	FWHM (°2θ)	Height	D-Spacing (Å)	Average D-Spacing (Å)	Crystallite Size (nm)	Avg Crystallite Size (nm)	Lattice Strain	Average Lattice Strain
EML0	Peak1	19.15	1.17	29.35	5.38		8.38		0.03	
	Peak2	20.94	2.76	200.38	4.92		3.55		0.07	
	Peak3	23.17	3.03	431.93	4.46		3.24		0.06	
	Peak4	24.00	0.76	105.14	4.30	4.43	13.04	8.06	0.02	0.04
	Peak5	24.98	0.95	300.49	4.14		10.36		0.02	
	Peak6	25.91	0.75	120.54	3.99		13.21		0.01	
	Peak7	27.05	2.12	178.91	3.83		4.67		0.04	
EML1	Peak1	19.51	1.22	42.17	5.28		8.02		0.03	
	Peak2	21.07	2.19	174.56	4.89		4.47		0.05	
	Peak3	23.13	2.68	372.29	4.46		3.67		0.06	
	Peak4	24.08	0.92	119.18	4.29	4.41	10.68	7.88	0.02	0.03
	Peak5	24.98	0.90	296.94	4.14		11.03		0.02	
	Peak6	25.83	0.79	131.11	4.00		12.61		0.01	
	Peak7	27.04	2.12	168.77	3.83		4.69		0.04	
EML2	Peak1	20.10	2.32	92.13	5.13		4.23		0.06	
	Peak2	22.59	3.40	383.84	4.57		2.90		0.07	
	Peak3	22.62	0.60	54.54	4.56		16.29		0.01	
	Peak4	24.22	1.78	173.35	4.27		5.54		0.04	
	Peak5	25.02	0.73	160.01	4.13	4.29	13.61	9.20	0.01	0.03
	Peak6	25.89	1.17	142.60	4.00		8.43		0.02	
	Peak7	26.89	0.56	36.79	3.85		17.78		0.01	
	Peak8	27.30	2.06	134.99	3.79		4.81		0.04	
EML3	Peak1	20.45	0.40	8.45	5.04		24.25		0.01	
	Peak2	21.90	1.95	110.59	4.71		5.04		0.04	
	Peak3	22.85	0.60	46.00	4.52		16.32		0.01	
	Peak4	23.89	2.73	236.51	4.32		3.62		0.06	
	Peak5	25.15	0.71	95.33	4.11	4.28	13.99	11.54	0.01	0.03
	Peak6	26.16	1.25	104.12	3.96		7.90		0.02	
	Peak7	27.07	0.66	44.56	3.82		14.98		0.01	
	Peak8	27.83	1.60	93.70	3.72		6.22		0.03	

**Table 3:** Composition of 20 g of Oleogels.

Sample	Emulsifier Present	SW (g)	RBO (g)	Emulsifier Stock (g)	Emulsifier Content (%)	Nutraceutical Content (g)
EML0	-	3	17	0	0	-
EML1	SAC	3	12	5	0.025	-
EML2	SFL	3	12	5	0.025	-
EML3	SPN	3	12	5	0.025	-
EML0	-	3	17	0	0	0.1
EML1N	SAC	3	12	5	0.025	0.1
EML2N	SFL	3	12	5	0.025	0.1
EML3N	SPN	3	12	5	0.025	0.1

## Data Availability

Not applicable.
